# Obesity Alters the microRNA Expression Profile Related to Metabolic Disorders in Peripheral Blood Mononuclear Cells: Preliminary Results

**DOI:** 10.3390/cimb47100799

**Published:** 2025-09-26

**Authors:** Samar Sultan, Marwah Maashi

**Affiliations:** 1 Medical Laboratory Sciences, Faculty of Applied Medical Sciences, King Abdulaziz University, Jeddah 21589, Saudi Arabia; mmaashi@kau.edu.sa; 2 Regenerative Medicine Unit, King Fahd Medical Research Center, King Abdulaziz University, Jeddah 21589, Saudi Arabia

**Keywords:** obesity, microRNAs, metabolic disorders, epigenetic

## Abstract

Obesity is a major global health issue associated with an increased risk of early-onset metabolic disorders and chronic inflammation. Identifying the epigenetic mechanisms that contribute to obesity-related metabolic and inflammatory dysregulation is crucial for developing effective prevention and treatment strategies. This pilot study aimed to investigate the effects of obesity on the expression of microRNAs (miRNAs) related to metabolic disorders in human peripheral blood mononuclear cells from metabolically healthy obese subjects and non-obese controls. Differentially expressed miRNAs in TaqMan human miRNA arrays were quantified using quantitative PCR. To validate the robustness and generalizability of our findings, we performed cross-validation using the publicly available GSE155096 dataset. The expression of miR-145-5p was significantly increased (4.913-fold change) in obese individuals compared to the non-obese control group. Two miRNAs, miR-27b-3p and miR-17-5p, were downregulated 2.207- and 1.448-fold, respectively, approaching significance. A positive correlation was established between miR-145-5p and free triiodothyronine, eosinophils, and vitamin D. A cross-validation analysis confirmed the direction of change for these key miRNAs. The data suggest that miR-145-5p, miR-27b-3p, and miR-17-5p could be implicated in the progression of obesity in causing metabolic abnormalities, clarifying how molecular factors cause the metabolic deregulation associated with obesity.

## 1. Introduction

Obesity is becoming an increasingly significant public health problem in the Kingdom of Saudi Arabia (KSA) and globally. A study carried out in 2023 indicated that 23% of the population in the KSA are classified as obese, as defined by a body mass index (BMI) of 30 or above [1]. Of particular concern is the growing number of overweight or obese adolescents in the KSA [2]. The literature extensively suggests that obese adults are increasingly facing obesity-related health challenges, such as atherosclerosis, metabolic syndrome, hypertension, and type 2 diabetes mellitus (T2D) [3]. Nonetheless, some individuals with an increased BMI do not develop metabolic syndrome and are typically referred to as “metabolically healthy obese” [4]. However, the underlying protective mechanisms remain unclear. Characteristics of obesity that may influence epigenetic regulatory processes include hyperglycemia, uncontrolled endocrine disruptors, hypoxia, oxidative stress, and inflammatory processes [5]. Interestingly, research indicates that adipocytes can retain obesity-induced epigenetic alterations, predisposing individuals to weight regain after caloric restriction and promoting inflammatory responses in the liver [6].

Researchers have identified numerous epigenetic mechanisms, including posttranslational modifications of histone proteins, DNA methylation, nucleosome spacing, chromatin structure, and the role of microRNAs (miRNAs) [7,8]. MiRNAs are small, non-coding RNA molecules typically 20 to 24 nucleotides long. They serve as regulators of gene activity at the post-transcriptional stage by attaching to complementary sequences on messenger RNA, facilitating its degradation or preventing translation into a protein. Numerous studies have explored the changes in miRNA expression in obesity. In their study, Mir et al. [9] detected differential expression of 64 miRNAs in blood samples, when comparing metabolically healthy obese individuals with metabolically unhealthy obese individuals. Furthermore, they linked miR-130b-5p, miR-363-3p, and miR-636 to cholesterol concentrations, while miR-130a-3p was found to be correlated with low-density lipoprotein (LDL) concentrations.

Another study found a unique signature of plasma miRNAs, comprising miR-375, miR-144-5p, miR-20a-3p, miR-145-5p, and miR-21-3p, which may improve cardiovascular risk monitoring in patients with severe obesity after bariatric surgery [10]. Brando et al. [11] compiled a list of key circulating miRNAs that are modified in individuals with obesity. Interestingly, certain miRNAs, such as miR-92a-3p, miR-122, miR-122-5p, miR-140-5p, miR-142-3p, miR-151a, miR-155, miR-222, and miR-15a, showed increased expression. In contrast, other miRNAs, including miR-15a, miR-26a, miR-30b, miR-30c, miR-125b, miR-126, miR-139-5p, miR-144-5p, miR-146a, miR-150, miR-223, and miR-376a, were expressed at lower levels in obese adults compared to healthy lean controls. Thus, the inconsistency in these results suggests that more studies are required to investigate miRNA expression levels in obesity to identify suitable biomarkers for preventing associated complications, particularly metabolic syndrome. Therefore, this pilot study aimed to explore how obesity influences the expression of miRNAs associated with metabolic disorders in human peripheral blood mononuclear cells obtained from metabolically healthy obese individuals and non-obese controls.

## 2. Materials and Methods

### 2.1. Ethical Approval

The research protocol was reviewed, and ethical approval was obtained from the Institutional Review Board (IRB), General Directorate of Health Affairs, Tabuk Region (registration no. H-07-TU-077, 24 May 2021). All participants provided written informed consent after being thoroughly informed of the procedures and objectives of the principal investigator.

### 2.2. Study Participants

This study employed a case–control design, enrolling six obese participants who were metabolically healthy and matching them by age with six healthy, non-obese controls. The participants were recruited between July 2021 and November 2021. The inclusion criteria were age 20–50 years, with obesity defined as a BMI > 30 kg/m^2^, according to the World Health Organization. Healthy individuals with BMI < 25 kg/m^2^ served as controls. For anthropometrics, height and weight measurements were obtained from patient medical records to calculate BMI (weight in kg/height in m^2^). All participants did not take any medications and did not have comorbidities.

### 2.3. Biochemical Tests

Each participant provided two blood samples: one was obtained in plain tubes to assess biochemical parameters, and the other was collected using ethylenediaminetetraacetic acid blood collection tubes to evaluate hematological parameters and RNA extraction. Assays were performed at the Maternity and Children’s Hospital in Tabuk. Biochemical parameters, such as glucose, BUN, creatinine, albumin, total protein, cholesterol, triglycerides, high-density lipoprotein, LDL, amylase, and lactate dehydrogenase (LDH), were assessed using colorimetric methods with the same analyzer. Serum levels of vitamin D, thyroid-stimulating hormone, thyroxine, and triiodothyronine (T3) were evaluated using a chemiluminescent immunoassay on a DXI 800 Beckman Coulter analyzer (Beckman Coulter, Brea, CA, USA). Complete blood count was performed using a Beckman Coulter DXH 900 (Beckman Coulter, Brea, CA, USA) hematology analyzer.

### 2.4. RNA Extraction and miRNA Detection

Following the manufacturer’s protocol, the miRNeasy Mini Kit (Qiagen, Hilden, Germany) was used to isolate total RNA, including small RNA species, from peripheral blood mononuclear cells. The concentration and purity of total RNA were assessed using a NanoDrop 2000 spectrophotometer (Thermo Fisher Scientific, Waltham, MA, USA). An absorbance ratio of 260 to 280 nm (A260/A280) within the range of 1.9–2.1 confirmed that the RNA samples were pure. Complementary DNA synthesis and TaqMan human miRNA arrays were quantified using quantitative PCR and performed as previously described [12].

### 2.5. Cross-Validation Procedure

To assess the robustness of our findings and the predictive performance of the model, we performed a cross-validation analysis. The dataset, comprising miRNA expression data and clinical variables, was subjected to a cross-validation analysis using Python version 3.13.7 software. We divided the data into training and testing sets. Training was performed on GSE155096 obesity data. (https://www.ncbi.nlm.nih.gov/gds/?term=GSE155096[Accession] (accessed on 15 September 2020)). Permutation Tests were performed for cross-validation of significance because the sample size was very small. The Area Under the ROC curve (AUC) was obtained for the trained models, and it was selected due to insensitivity to class imbalance. The Regression Model was trained to find correlation between significant miRNAs expression and clinical data adjusting for age and BMI.

It is important to note that due to the relatively small sample size, cross-validation results should be interpreted cautiously, as they are susceptible to over-fitting. Despite this limitation, the statistical report showed a high AUC of 0.88 in the nested cross-validation. This score suggests a good discriminatory power of the model to classify obesity, yet the need for the validation of the results in different populations with larger sample sizes is a key factor that must be considered in their interpretation.

The Regression Model was trained to find correlations between the expression of significant miRNAs and clinical data adjusting for age and BMI.

### 2.6. Statistical Analysis

Relative quantifications, tables, and relative expression box plots were generated using Microsoft Excel. Relative quantification was performed via the 2−ΔΔCt method. Clinical data were analyzed using the Shapiro–Wilk normality test, median, interquartile range, mean, standard deviation, and two-tailed unpaired *t*-test. Multiple linear regression was used to assess correlation. Under the assumptions of non-normality and limited sample size, both the Mann–Whitney U test and the Permutation Test were used for cross-validation of significance. Statistical significance was set at *p* < 0.05.

## 3. Results

### 3.1. Patient Characteristics

As shown in Table 1, the experimental group had significantly higher weight and BMI (*p* < 0.001) than the control group. No significant differences were observed in the other clinical variables.

### 3.2. MiRNA Relative Expression Changes

**Figure 1:**
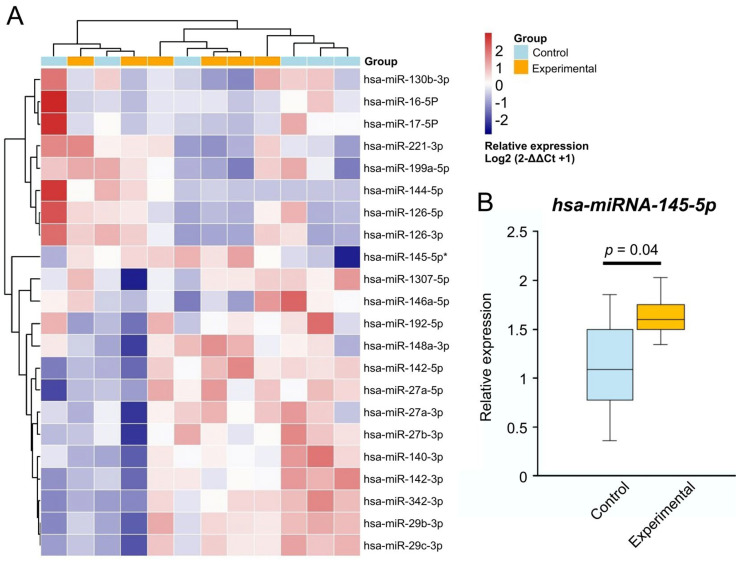
Expression changes in obesity-related miRNAs. (**A**) MiRNA expression heatmap. Relative gene expression in log2(2−ΔΔCt + 1) showing hierarchical clusters. Red indicates upregulation, while blue indicates downregulation. The asterisk shows the significant *p* < 0.05. (**B**) Control (blue) vs. experimental (orange) hsa-miRNA-145-5p expression box plot. Two-tailed unpaired *t*-test.

Some of the 28 analyzed miRNAs were excluded due to undetectable expression, leaving 22 miRNAs for further analysis. The relative gene expression heatmap (Figure 1A) revealed marked differences between a portion of the control and experimental miRNA expression results, clustering separately, suggesting that miRNAs may be affected by the experimental conditions.

Hsa-miRNA-145-5p was the only significantly upregulated miRNA in the obese group compared to the control group (Figure 1B, 4.913-fold change, *p* = 0.04). MiR-27b-3p and miR-17-5P were downregulated (−2.207- and −1.448-fold change, respectively), approaching statistical significance (*p* = 0.07 and *p* = 0.09, respectively).

### 3.3. Correlation Between miRNA Expression and Clinical Parameters

**Figure 2:**
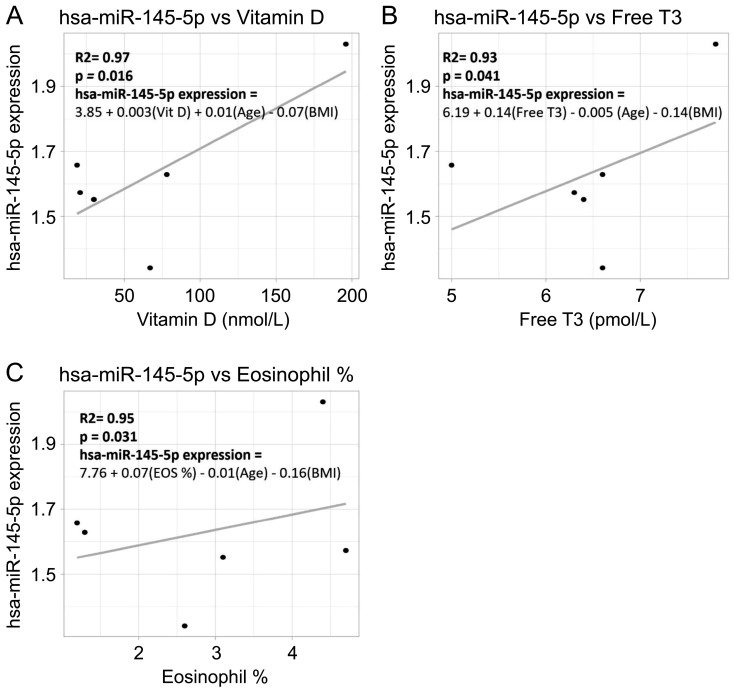
Significant correlations between hsa-miR-145-5p and clinical features. Scatterplots showing correlation between hsa-miR-145-5p relative gene expression vs. (**A**) vitamin D, (**B**) free triiodothyronine, and (**C**) eosinophil percentage. The multiple linear regression equation for hsa-miR-145-5p adjusted for age and body mass index is shown in the graph. *p* indicates statistical significance (*p* < 0.05).

Clinical data was adjusted for age and BMI using a multiple linear regression model. hsa-miR-145-5p was significantly positively correlated with levels of vitamin D (Figure 2A), free T3 (Figure 2B), and eosinophil percentage (Figure 2C, R2 > 0.90, *p* < 0.05).

### 3.4. Cross-Validation Using Open-Source Datasets

We used the GSE155096 dataset to cross-validate our miRNA expression data and assess the consistency of the observed fold change direction. Table 2 shows the following: Up-regulated miRNAs in obesity: hsa-miR-145-5p, hsa-miR-27a-5p, hsa-miR-142-5p. Obesity enhances vascular protective, metabolic, and immune-regulatory pathways that may counteract obesity-associated dysfunction.

Down-regulated miRNAs in obesity: hsa-miR-342-3p, hsa-miR-142-3p, hsa-miR-27b-3p, hsa-miR-27a-3p, hsa-miR-140-3p, hsa-miR-130b-3p, hsa-miR-126-5p, hsa-miR-192-5p, hsa-miR-17-5p, hsa-miR-16-5p. Many of these are linked to adipogenesis, lipid metabolism, cell proliferation, and chronic inflammation, suggesting that obesity suppresses pathways that promote fat accumulation and metabolic stress. No consistent change: hsa-miR-29b-3p, hsa-miR-29c-3p, hsa-miR-148a-3p, hsa-miR-146a-5p, hsa-miR-221-3p. These miRNAs show no meaningful difference, indicating they may not play a central role in the obesity response.

**Figure 3:**
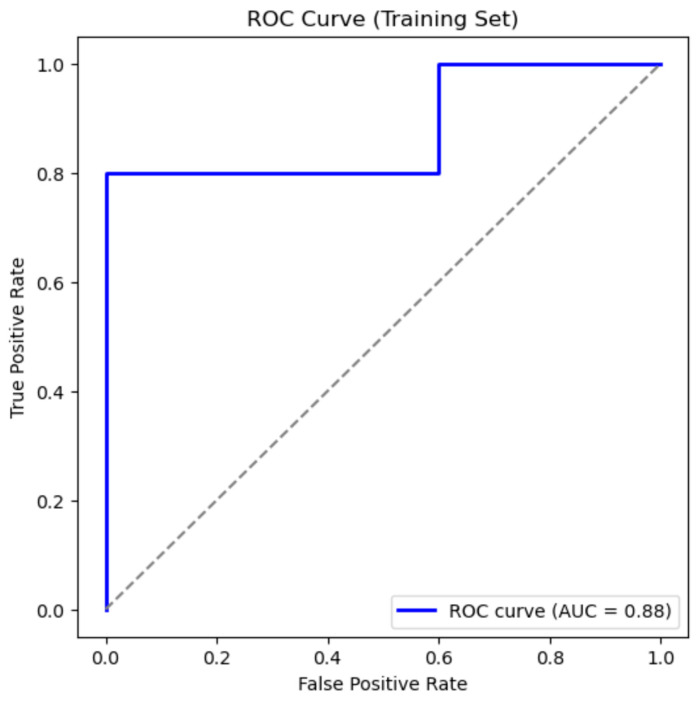
Cross Validation for Model using ROC Curve (Training set).

The receiver operating characteristic (ROC) curve analysis, performed as part of the cross-validation assessment, demonstrated strong discriminative ability of the model (Figure 3). The area under the ROC curve (AUC) was 0.88, indicating that the model effectively distinguished between the training data points with obesity and those without.

#### 3.4.1. Model Reliability and Accuracy Results

As depicted in Table 3, our model did a good job of telling apart healthy individuals from those with obesity. For the healthy group, it was very accurate: when the model predicted that someone was healthy, it was correct most of the time (precision = 0.83), and it caught every single healthy case (recall = 1.0), leading to a strong overall score (F1 = 0.91). 

For the obesity group, the model was correct when it predicted obesity (precision = 1.0), meaning there were no false positives. It did miss a few true obesity cases (recall = 0.80), but overall performance remained high (F1 = 0.89).

#### 3.4.2. The Regression Model

Our integrative analysis combining clinical parameters with miRNA expression data (trained on the GSE155096 obesity dataset) revealed several significant associations that highlight the interplay between lipid metabolism, endocrine function, and immune regulation in obesity-related metabolic syndrome, as shown in Table 4. 

## 4. Discussion

Obesity is recognized as a multifactorial condition marked by the accumulation of fat mass and elevated BMI, which is associated with metabolic disorders [13]. MiRNAs are key regulators of metabolic balance [14]. In this study, individuals with obesity exhibited alterations in miRNAs related to metabolic disorders, including increased expression of miR-145-5p, which was associated with the levels of free T3, eosinophils, and vitamin D. Obese subjects also exhibited low expression of miR-27b-3p and miR-17-5p, approaching significance.

MiR-145 inhibits fat breakdown by suppressing target proteins FOXO1 and ABHD5 [15]. Similar to our current finding regarding miR-145-5p, elevated levels of miR-145-5p expression have been reported in individuals with severe obesity, which diminished following bariatric surgery [16]. However, the role of miR-145-5p in obesity and CVD remains unclear [17,18]. Enhanced hypoxia and production of reactive oxygen species have been associated with miR-145-5p expression [19], which exhibited a significant positive correlation with brain-derived neurotrophic factor, known for its protective role against oxidative stress in ischemic tissues [20]. The authors suggested that increased levels of miR-145-5p may be linked to oxidative stress in severe obesity prior to bariatric surgery, as previously reported [21]. The authors suggested that this reduction could reflect the improvement observed after bariatric surgery. Jordan et al. [22] discovered that the expression levels of miR-143 and miR-145 increased in a mouse model of diet-induced obesity.

In contrast, the expression levels of miR-145-5p and miR-143-3p are notably diminished in subcutaneous white adipose tissue in cases of obesity and insulin resistance [23]. Reduced miR-145 expression is associated with liver inflammation, whereas its overexpression suppresses inflammation by inhibiting the proinflammatory transcription factor nuclear factor kappa B [24]. The authors suggested that increasing miR-145 levels could represent a novel therapeutic approach for the prevention and treatment of metabolic disorders, including T2D and atherosclerosis [24]. This implies that, in the current study, the increased expression of miR-145-5p in metabolically healthy obese individuals might block the inflammatory environment with the progression of metabolic disorders; however, this needs further investigation.

In the current study, a positive correlation was found between miR-145-5p and free T3, eosinophils, and vitamin D. These correlations provide a theoretical basis for research into definitive causal links. Further studies are needed to understand the relationship with free T3, which could possibly indicate an association of thyroid function with obesity via miR-145-5p. One study identified miR-145-5p as the most significantly downregulated miRNA in patients with recurrent thyroid cancer [25]. MiR-145-5p has been implicated in cancer and asthma, which could explain the observed relationship with eosinophil percentage [26]. This miRNA also has a complex association with asthma and vitamin D [27]. Interestingly, obese patients with T2D who are deficient in vitamin D exhibit elevated levels of miR-143 and miR-145 expression [28]. The authors suggested vitamin D status assessment is crucial in patients with T2D to enhance clinical outcomes during treatment [28]. Thus, elevated levels of miR-143 and miR-145 may serve as predictive markers of obesity-related T2D. These initial correlations are intriguing and may provide a basis for further investigation into the role of miR-145-5p in metabolic regulation and immune function in obesity.

Notably, in the current study, a non-significant downregulation of miR-27b-3p and miR-17-5p was observed in obese individuals compared to controls. Inhibition of miR-27b-3p promotes the browning of epididymal fat in mice and induces obesity in high-fat diet [29]. Another study revealed that miR-27a/b work together to inhibit adipose thermogenesis and encourage metabolic dysfunction in obesogenic environments. Therefore, targeting the miR-27a/b pathway could provide a new therapeutic strategy to increase energy expenditure and address metabolic diseases associated with obesity [30].

One study showed that miR-17-5p regulates glucose balance by influencing the downstream target CREB, an essential protein involved in glucose metabolism and adipocyte activity [31]. A statistically significant reduction was found in the levels of circulating miR-17-5p and miR-132 in obese individuals compared to healthy controls [16]. Research has shown that the abnormal expression of miRNAs and their target genes plays a crucial role in the development of cardiometabolic disease [32]. Thus, the mechanistic links proposed between miR-145-5p, miR-27b-3p, and miR-17-5p suggest that these are part of a complex regulatory network that governs adipocyte development, inflammation, and metabolic homeostasis in obesity [33].

To validate our findings, we performed cross-validation using the publicly available GSE155096 dataset. While acknowledging the limitations imposed by the sample size and experimental design of GSE155096, the analysis revealed a consistent trend towards upregulation of miR-145-5p and downregulation of miR-27b-3p and miR-17-5p in obese individuals, mirroring the directional changes observed in our data. These preliminary cross-validation results, although not definitive due to inherent limitations, inform future research by highlighting the potential for these miRNAs to serve as biomarkers of obesity-related metabolic dysregulation. A targeted analysis with a larger, more homogenous dataset could refine these preliminary correlations

Cross-validation regression analysis demonstrated a consistent negative correlation between a panel of miRNAs (miR-140-3p, miR-192-5p, miR-27a-3p, miR-27a-5p, miR-27b-3p, miR-342-3p) and lipid markers (cholesterol and LDL levels) [34], suggesting that obesity-induced dyslipidemia is accompanied by suppression of miRNAs known to participate in vascular protection and lipid homeostasis. In addition, miR-142 family members showed strong immune-related associations, with miR-142-5p positively linked to monocytes and negatively to neutrophils, underscoring its role in the regulation of inflammatory cell balance during obesity [35]. Endocrine associations were also observed, where miR-27a-5p correlated positively with TSH and negatively with free T3, while miR-142-5p was inversely associated with free T4, highlighting a potential regulatory axis between thyroid hormones and miRNAs in energy metabolism. Furthermore, miR-142-3p showed an inverse association with creatinine, pointing to links with renal stress and systemic metabolic imbalance. Taken together, these results suggest that miRNAs identified in GSE155096 not only distinguish obesity status but also track clinically relevant metabolic and endocrine disturbances, strengthening their potential as biomarkers and mechanistic mediators in obesity-related metabolic syndrome.

The main limitation of this study was the small number of significant miRNAs, which could be due to the small sample size, as noted for similar studies in the literature [36,37,38]. While the sample size is small, the findings are consistent with existing literature supporting their potential relevance. Thus, the current findings must be interpreted with caution. This study was designed as a preliminary pilot investigation, primarily aimed at assessing the feasibility of detecting differential miRNA expression related to obesity in human peripheral blood mononuclear cells. The primary goal was to generate hypotheses and identify candidate miRNAs, such as miR-145-5p, miR-27b-3p, and miR-17-5p, that may play roles in the molecular mechanisms linking obesity to metabolic dysregulation. Despite the limited cohort, we observed notable changes and correlations that suggest potential biological relevance. We recommend that future research expands on this work by including larger, more diverse populations to enhance statistical robustness, validate these preliminary findings, and better understand the clinical implications. This will help in the prevention and formulation of strategies to address metabolic complications associated with obesity. Another limitation is that we lacked information from donors regarding their lifestyle factors, blood pressure, and insulin resistance markers, which could potentially affect miRNA expression.

## 5. Conclusions

This study suggests that miR-145-5p, miR-27b-3p, and miR-17-5p may play a role in obesity development and its progression to metabolic disorders. Consistent miRNA expression signatures across independent datasets strengthen their potential as biomarkers for obesity-related metabolic dysregulation, providing insights into underlying molecular mechanisms.

## Figures and Tables

**Table 1:** Clinical data of participants.

Parameter	Control		Obese		* *p* Value
Gender Male/Female	5/1		3/3		-
	Mean	± SD	Mean *	± SD	
Age (years)	27.67	5.79	33.67	9.61	0.22
Height (cm)	163.33	12.68	162.17	9.37	0.86
Weight (kg)	59.17	10.40	98.33	12.11	0.00013
BMI	22.05	1.42	37.28	1.49	0.000001
GLU (mmol/L)	5.06	0.71	5.31	0.65	0.54
BUN (nmol/L)	4.0	0.85	4.09	0.81	0.85
Creatinine (µmol/L)	69	16	68.60	13	0.97
Uric acid (µmol/L)	313	73	338	99	0.64
Cholesterol (mmol/L)	5.10	0.79	5.52	1	0.46
TRG (mmol/L)	0.90	0.37	1.38	0.56	0.11
HDL (mmol/L)	1.58	0.27	1.46	0.34	0.48
LDL (mmol/L)	3.38	0.71	3.71	0.84	0.48
Albumin (g/L)	43	1.97	43.28	2.79	0.87
Total protein (g/L)	72.45	3.18	72.23	3.60	0.72
LDH (U/L)	188	38	166	34.60	0.34
Amylase (U/L)	73	22	67.30	19	0.64
TSH (µmol/L)	1.62	0.90	2.39	1.18	0.23
Free T3 (pmol/L)	6.17	0.76	6.45	0.89	0.57
Free T4 (pmol/L)	12.33	1.2	12.33	2	1
Vitamin D (nmol/L)	62.50	27	68.50	0.7	0.84
WBC (10^9^/L)	6.48	1.2	6.68	2	0.87
Platelet (10^9^/L)	275	51	279	37	0.88

* Independent sample *t*-test.

**Table 2:** Significant miRNAs by Fold change (qPCR).

miRNA	U-Statistic	*p*-Value	adj_pval	Mean Control	Mean-Obesity	Fold-Change (Obesity/Control)	Direction
hsa-miR-145-5p	36	0.002	0.007	1.11	1.63	1.5	in Obesity
hsa-miR-29b-3p	36	0.002	0.007	1.19	1.16	1	no change
hsa-miR-29c-3p	36	0.002	0.007	1.14	1.11	1	no change
hsa-miR-342-3p	36	0.002	0.007	1.33	1.09	0.82	in Obesity
hsa-miR-27a-5p	36	0.002	0.007	1.42	1.9	1.3	in Obesity
hsa-miR-142-5p	36	0.002	0.007	1.15	1.47	1.3	in Obesity
hsa-miR-142-3p	36	0.002	0.007	1.2	0.91	0.75	in Obesity
hsa-miR-1307-5p	34	0.009	0.021	1.05	0.96	0.9	no clear trend
hsa-miR-27b-3p	34	0.009	0.021	1.03	0.73	0.71	in Obesity
hsa-miR-27a-3p	34	0.009	0.021	1.08	0.91	0.84	in Obesity
hsa-miR-148a-3p	33	0.015	0.030	1.08	1.06	1	no change
hsa-miR-140-3p	33	0.015	0.030	1.06	0.78	0.74	in Obesity
hsa-miR-146a-5p	32	0.026	0.045	1.05	0.99	0.9	no change
hsa-miR-221-3p	31	0.041	0.062	1.08	1.13	1	no change
hsa-miR-130b-3p	31	0.041	0.062	1.12	0.88	0.79	in Obesity
hsa-miR-126-5p	30	0.065	0.078	1.45	1.16	0.80	in Obesity
hsa-miR-192-5p	30	0.065	0.078	1.37	0.86	0.63	in Obesity
hsa-miR-17-5P	30	0.065	0.078	1.3	0.62	0.53	in Obesity
hsa-miR-16-5P	30	0.065	0.078	1.29	0.55	0.43	in Obesity

**Table 3:** Model Reliability and accuracy.

	Precision	Recall	F1-Score
Healthy	0.83	1	0.91
Obesity	1	0.8	0.89

**Table 4:** Regression model to find correlations between significant miRNAs expression and obesity clinical data adjusting for age and BMI.

miRNA	Clinical Variable	Regression Model	Adjusted R2	*p*-Value	Significant
hsa-miR-140-3p	CHOL	hsa-miR-140-3p = 3.107 − 0.274*CHOL − 0.018*Age − 0.006*BMI	0.700	0.003	Yes
hsa-miR-140-3p	LDL	hsa-miR-140-3p = 2.776 − 0.340*LDL − 0.015*Age − 0.007*BMI	0.726	0.002	Yes
hsa-miR-142-3p	CREA	hsa-miR-142-3p = 3.806 − 0.023*CREA − 0.018*Age − 0.026*BMI	0.595	0.019	Yes
hsa-miR-142-3p	FREE_T3	hsa-miR-142-3p = 4.639 − 0.356*FREE_T3 − 0.037*Age − 0.011*BMI	0.515	0.043	Yes
hsa-miR-142-5p	FREE_T4	hsa-miR-142-5p = 5.042 − 0.291*FREE_T4 − 0.040*Age + 0.037*BMI	0.579	0.011	Yes
hsa-miR-142-5p	MONO	hsa-miR-142-5p = − 0.088 + 0.261*MONO − 0.070*Age + 0.043*BMI	0.568	0.012	Yes
hsa-miR-142-5p	NEUT	hsa-miR-142-5p = 4.226 − 0.040*NEUT − 0.071*Age + 0.040*BMI	0.412	0.047	Yes
hsa-miR-192-5p	CHOL	hsa-miR-192-5p = 3.991 − 0.479*CHOL − 0.010*Age − 0.004*BMI	0.555	0.005	Yes
hsa-miR-192-5p	LDL	hsa-miR-192-5p = 3.345 − 0.567*LDL − 0.004*Age − 0.007*BMI	0.519	0.008	Yes
hsa-miR-27a-3p	CHOL	hsa-miR-27a-3p = 2.651 − 0.252*CHOL − 0.019*Age + 0.008*BMI	0.527	0.011	Yes
hsa-miR-27a-3p	LDL	hsa-miR-27a-3p = 2.321 − 0.303*LDL − 0.015*Age + 0.007*BMI	0.513	0.012	Yes
hsa-miR-27a-5p	CHOL	hsa-miR-27a-5p = 4.415 − 0.676*CHOL − 0.030*Age + 0.058*BMI	0.315	0.037	Yes
hsa-miR-27a-5p	FREE_T3	hsa-miR-27a-5p = 6.079 − 0.799*FREE_T3 − 0.038*Age + 0.060*BMI	0.348	0.030	Yes
hsa-miR-27a-5p	LDL	hsa-miR-27a-5p = 3.466 − 0.788*LDL − 0.021*Age + 0.054*BMI	0.274	0.048	Yes
hsa-miR-27a-5p	TSH	hsa-miR-27a-5p = 1.125 + 0.673*TSH − 0.035*Age + 0.009*BMI	0.438	0.016	Yes
hsa-miR-27b-3p	LDL	hsa-miR-27b-3p = 2.292 − 0.211*LDL − 0.013*Age − 0.009*BMI	0.528	0.035	Yes
hsa-miR-342-3p	CHOL	hsa-miR-342-3p = 4.916 − 0.487*CHOL − 0.053*Age + 0.017*BMI	0.515	0.023	Yes
hsa-miR-342-3p	LDL	hsa-miR-342-3p = 4.305 − 0.594*LDL − 0.046*Age + 0.014*BMI	0.520	0.022	Yes

CHOL: Cholesterol; LDL: low-density lipoprotein; TSH: Thyroid stimulating hormone; NEUT: Neutrophil; MONO: Monocyte; CREA: Creatinine

## Data Availability

The data that support the findings of this study are available in the figshare repositories at (https://doi.org/10.6084/m9.figshare.30015478.v1, 29 August 2025).

## Abbreviations

The following abbreviations are used in this manuscript:

miRNAs	microRNAs
KSA	Kingdom of Saudi Arabia
BMI	body mass index
T2D	type 2 diabetes mellitus
LDL	low-density lipoprotein
IRB	Institutional Review Board
LDH	lactate dehydrogenase
T3	Triiodothyronine
